# A Systems Biology Approach to Characterize the Regulatory Networks Leading to Trabectedin Resistance in an *In Vitro* Model of Myxoid Liposarcoma

**DOI:** 10.1371/journal.pone.0035423

**Published:** 2012-04-16

**Authors:** Sarah Uboldi, Enrica Calura, Luca Beltrame, Ilaria Fuso Nerini, Sergio Marchini, Duccio Cavalieri, Eugenio Erba, Giovanna Chiorino, Paola Ostano, Daniela D'Angelo, Maurizio D'Incalci, Chiara Romualdi

**Affiliations:** 1 Department of Oncology, Mario Negri Institute for Pharmacological Research, Milan, Italy; 2 Department of Biology, University of Padova, Padova, Italy; 3 Department of Computational Biology, Research and Innovation Centre, San Michele all'Adige, Trento, Italy; 4 Fondazione Edo ed Elvo Tempia Valenta, Cancer Genomics Laboratory, Biella, Italy; 5 Experimental Endocrinology and Oncology, CNR, Naples, Italy; University of Turin, Italy

## Abstract

Trabectedin, a new antitumor compound originally derived from a marine tunicate, is clinically effective in soft tissue sarcoma. The drug has shown a high selectivity for myxoid liposarcoma, characterized by the translocation t(12;16)(q13; p11) leading to the expression of FUS-CHOP fusion gene. Trabectedin appears to act interfering with mechanisms of transcription regulation. In particular, the transactivating activity of FUS-CHOP was found to be impaired by trabectedin treatment. Even after prolonged response resistance occurs and thus it is important to elucidate the mechanisms of resistance to trabectedin. To this end we developed and characterized a myxoid liposarcoma cell line resistant to trabectedin (402-91/ET), obtained by exposing the parental 402-91 cell line to stepwise increases in drug concentration. The aim of this study was to compare mRNAs, miRNAs and proteins profiles of 402-91 and 402-91/ET cells through a systems biology approach. We identified 3,083 genes, 47 miRNAs and 336 proteins differentially expressed between 402-91 and 402-91/ET cell lines. Interestingly three miRNAs among those differentially expressed, miR-130a, miR-21 and miR-7, harbored CHOP binding sites in their promoter region. We used computational approaches to integrate the three regulatory layers and to generate a molecular map describing the altered circuits in sensitive and resistant cell lines. By combining transcriptomic and proteomic data, we reconstructed two different networks, *i.e.* apoptosis and cell cycle regulation, that could play a key role in modulating trabectedin resistance. This approach highlights the central role of genes such as CCDN1, RB1, E2F4, TNF, CDKN1C and ABL1 in both pre- and post-transcriptional regulatory network. The validation of these results in *in vivo* models might be clinically relevant to stratify myxoid liposarcoma patients with different sensitivity to trabectedin treatment.

## Introduction

Trabectedin (Yondelis®) is a tetrahydroisoquinoline molecule originally derived from the Caribbean marine tunicate, *Ecteinascidia turbinata*, with peculiar *in vitro* and *in vivo* cytotoxic activity in a wide range of tumor type: it has been approved by EMEA for the second line therapy of soft tissue sarcomas and ovarian cancer [1]–[7].

From a mechanistic point of view, trabectedin binds to the minor groove of DNA, differently from the conventional alkylating agents, forming DNA adducts on N_2_ position of guanine, and bends DNA towards the major groove; two of the three fused rings of this molecule are involved in the minor groove binding while the third (ring C) plays an important role in the process of interaction with different DNA-binding proteins [8], [9]. It is now widely accepted that trabectedin-DNA interaction affects transcription regulation although the level of selectivity is not completely elucidated [8], [10], [11].

Although trabectedin has shown activity against several types of sarcomas, its higher activity has been observed in patients with myxoid liposarcoma (MLS).

The high sensitivity displayed by MLS tumors is probably correlated to the ability of trabectedin to block the aberrant transcription activity of FUS-CHOP, the chimeric protein hallmark of MLS, generated by the chromosomal translocation t(12;16)(q13;p11) [12], [13].

Despite its activity, it is becoming a clinical experience that patients with long lasting trabectedin treatment develop resistance over time. To our knowledge there are only few studies focused on the mechanism of resistance and even fewer are based on a systems biology approach.

To investigate the molecular pathways involved in trabectedin resistance, the 402-91/ET cell line, resistant to trabectedin, was generated from the parental type I myxoid liposarcoma cell line 402-91 by stepwise increasing drug concentrations [14]. Based on the assumption that trabectedin impairs mechanisms of transcription regulation, we exploited several high-throughput technologies to combine different layers of regulation to elucidate the molecular pathways involved in the mechanism of trabectedin resistance in a well defined *in vitro* model of myxoid liposarcoma, previously described [14].

## Materials and Methods

### Cells and experimental design

The MLS type I 402-91 cell line was kindly supplied by Dr. P. Aman [15], while the trabectedin resistant 402-91/ET cell line was obtained in our laboratory as previously described [14]. For the array experiments, different batches of exponentially growing 402-91 and 402-91/ET cells were seeded and then total RNA enriched in microRNA (miRNA) fraction and protein lysates were harvested according to standard protocols. Gene, miRNA and protein expression profiles were generated from three independent biological replicates. Each experiment was performed using four technical replicates.

### RNA purification and quantitative Reverse Transcription PCR (qRT-PCR)

Total RNA enriched in miRNA fraction was purified using the miRNeasy Mini Kit according to the manufacturer's instructions (Qiagen, Milan, Italy). RNA was quantified by spectrophotometry (Nanodrop; Thermo Scientific, Wilmington, USA) and quality assessed by on-chip electrophoresis (Bioanalyser RNA 6000 Nano, Agilent Technologies, Palo Alto, USA). Mature miRNA and mRNA expression levels were examined by qRT-PCR using Applied Biosystems 7900HT. Briefly, cDNA was generated from 1 µg of purified total RNA using the miScript® Reverse Transcription Kit, following the manufacturer's instructions (Qiagen). miRNA expression was quantified using QuantiTect SYBR Green PCR Master Mix and the specific miScript® Primer Assay (Qiagen); RU-6b was used as reference gene. mRNA expression was analyzed using QuantiFast SYBR Green PCR Master Mix (Qiagen) and dedicated primers that were designed as described [16]; cyclophilin A was used as reference gene. Experiments were run in triplicate for each case to assess technical variability. Analysis was performed by using the 2^−ΔΔCt^ protocol and the median values were compared using the non parametric Mann-Whitney *t*-test. Differences were considered statistically significant with a two sided *p-value*<0.05. All tests and data plots were done using GraphPad Prism Version 5.01 (GraphPad Software, La Jolla, Ca, USA).

**Figure 1:**
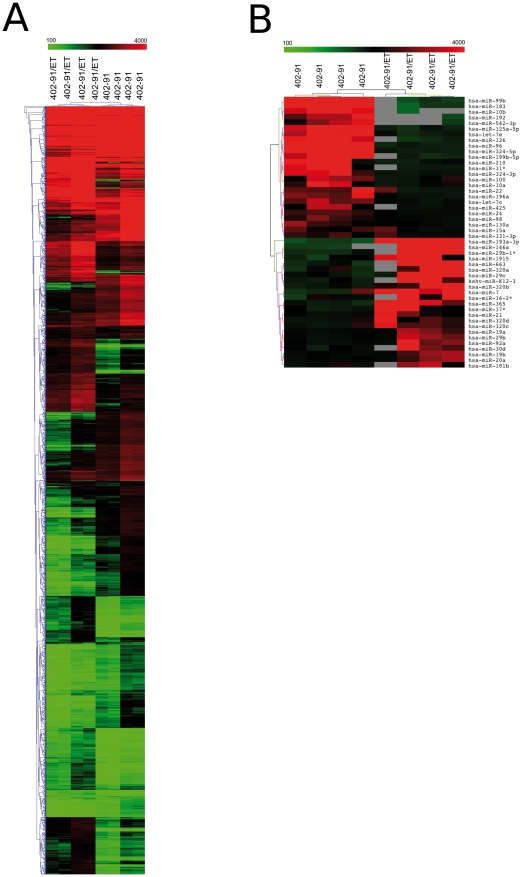
Heat maps of genes and miRNAs differentially expressed between 402-91/ET and 402-91 cells. **Panel A**. Heat map and cluster analysis of the 3,083 differentially expressed probes between resistant and sensitive cell lines. Red and green represent respectively differentially upregulated and downregulated genes in 402-91/ET cell lines. **Panel B**. Heat map and cluster analysis of the 48 miRNA differentially expressed between resistant and sensitive cell lines. Red and green represent respectively differentially upregulated and downregulated genes in 402-91/ET cell lines. Gray is for missing values.

### Western blot analysis

Total protein extracts were prepared by lysing cell extracts in lysis buffer (50 mM Tris-HCl, pH 8.0, 1% Triton X-100, 150 mM NaCl, 1 mM EGTA, 100 mM NaF, 10% glycerol (100%) and 1 mM MgCl_2_) with 2 mM phenylmethanesulfonyl fluoride (Sigma Aldrich, St. Louis, MO) as protease inhibitor, in agitation for 30 minutes at 4°C. Insoluble material was pelleted at 13 000 rpm at 4°C and the total protein concentration was determined by Bradford assay. The cellular proteins (40 µg) were separated using 8% and 15% (for detection of HMGA2 protein) SDS-PAGE and transferred electrophoretically to polyvinylidenedifluoride membranes (GE Healthcare, CA, USA). Blots were probed at 4°C overnight with primary antibodies in 5% milk/Tris Buffer Saline Tween20 (TBST). Immunoblotting was carried out with Cyclin D1 (Pharmingen, BD, La Jolla, CA, USA- 554181), E2F5 (abcam, Cambridge, UK - ab59769), SEMA4C (R&D Systems, Minneapolis MN, USA – AF6125), HMGA2 (kindly provided by Prof. Alfredo Fusco, Institute of Experimental Endocrinology and Oncology, CNR Naples, Italy) and PDCD4 (Sigma-Aldrich – SAB1407349) antibodies. Lamin B (Santa Cruz Biotechnology – sc6217) and Actin (Santa Cruz Biotechnology, CA, USA– sc130301) were used for loading control. Antibody binding was revealed by peroxidase labelled secondary antibodies and visualized using enhanced chemiluminescent (ECL) substrate (SuperSignal* West Pico, Thermo Scientific, Rockford IL USA). Experiments were repeated twice.

### Gene expression analysis

Gene expression (GE) was analyzed using Agilent slides on which 44,000 oligonucleotides were spotted (Agilent Technologies, Santa Clara, USA). Experiments were performed in four replicates, with dye-swapping duplication-scheme. The arrays were washed and scanned with a laser confocal scanner (G2565BA, Agilent Technologies) according to manufacturer's instructions. Oligo microarrays underwent standard post hybridization processing and the intensities of fluorescence were calculated by Feature Extraction software v.11 (Agilent Technologies). The microarray raw data are deposited in GEO (experiment number GSE20350) according to MIAME (Minimum Information About a Microarray Experiment) guidelines. GE signals were filtered according to the signal-to-background flag; features with more than 60% of signal below the local background were removed from the analysis. Then, lowess normalization was applied [17]. The identification of differentially expressed genes was performed using Empirical Bayes test [18] implemented in Bioconductor package limma in R software (http://www.r-project.org) with Benjamini and Hochberg False Discovery Rate (FDR) [19] to control for multiple testing problem. Given the large number of differentially expressed genes, we decided to adopt a more conservative strategy with respect to that applied for differentially expressed miRNAs and proteins: FDR≤0.01 and log fold change greater/lower than 1/-1 were considered to select differentially expressed features. Enrichment analysis of Gene Ontology (GO) categories and KEGG pathways was performed with DAVID web tool [20]. Cytoscape 2.7 [21] was used to reconstruct miRNA-target network.

Transcription factor binding site enrichment analysis was performed using oPOSSUM web tool [22].

### miRNA expression analysis

100 ng of total RNA enriched in miRNA fraction was Cy5-labelled and hybridized with a miRNA labelling and hybridization kit according to the manufacturer's instructions (Agilent Technologies). We used the commercially available G4470B human miRNA microarray kit (Agilent Technologies), which consists of 15 K features printed in an 8-plex format (8 of 15 K features printed in known human miRNAs :723 human and 76 human-viral miRNAs) sourced from the Sanger miRBASE public database, release 10.1. The arrays were washed and scanned (with a laser confocal scanner) according to the manufacturer's instructions. miRNA microarrays underwent standard post hybridization processing and the intensities of fluorescence were calculated by Feature Extraction software version 11 (Agilent Technologies). miRNA expression values were filtered as described in the previous section, and vsn algorithm has been applied to normalize the data [23]. miRNA data have been submitted to Array Express (accession number E-MTAB-965).

The identification of differentially expressed miRNAs has been performed using Empirical Bayes test [18] implemented in Bioconductor package limma of R software with Benjamini and Hochberg FDR to control for multiple testing problem. FDR≤0.1 were considered to select significant miRNAs.

mirandaSVR [24] was used to select putative miRNA target genes, while integration of mRNA and miRNA data was performed using MAGIA [25] web tool and dedicated in-house software.

Enrichment analysis of GO categories and KEGG pathways [26] of differentially expressed miRNA targets was performed with DAVID web tool [20] and MetaCore software [27].

CHOP transcription factor binding site identification on putative miRNA promoter regions was performed using miRGen2.0 [28] and CircuiDB database [29]. Cytoscape 2.7 [21] was used to reconstruct miRNA-target regulatory networks. KEGG database has been used to retrieve gene-gene interactions.

### Antibody microarray analysis

The Panorama Ab Microarray–XPRESS kit (Sigma-Aldrich) used in this study contains 725 different antibodies, spotted in duplicate on nitrocellulose-coated glass slides. These antibodies represent families of proteins known to be involved in a variety of different biological pathways (www.sigma.com/arrays). All reagents and solutions for protein purification were supplied within the kit and experiment was performed on four independent biological replicates, according to manufacturer's instructions. Briefly, total cell lysates were obtained from almost 10 million of exponentially growing 402-91 and 402-91/ET cells. 10 mg of total protein lysate, measured using Bradford protocol, were labeled with Cy3 and Cy5 fluorescent dyes (Amersham Biosciences, Buckinghamshire, UK) and their specific activity and concentration measured at the Nanodrop (Nanodrop, Thermo Scientific) after affinity column purification, provided within the kit. 150 µg of Cy3 and Cy5 labeled samples were co-hybridized on the same slides for 45′ at room temperature, accordingly to manufacturer's instructions. Washing and scanning (GENEPIX, version B) was performed according to the manufacturer's instructions. To increase the robustness of data analysis we performed technical replicates using dye-swap.

Scanned data files were first background subtracted using the normexp method [30]. Positive and negative control spots were removed, and the data were normalized using a modified rank-invariant selection algorithm (in-vMod) [31].

Determination of differential proteins between the two cell lines was carried out with linear models for microarray analysis [30], correcting the resulting p-value for multiple testing with FDR. To balance statistical significance and biological relevance, given the relatively low number of spots on the arrays, proteins were selected as differential expressed when their adjusted p-value was less than or equal to 0.1, and they were subsequently annotated with the EntrezGene and RefSeq identifiers of the corresponding genes to facilitate data integration.

Network analysis andGO enrichment of differential proteins was performed using the MetaCore software [27], using both public data and internal MetaCore curated maps, and with DAVID web tool [20]. Networks and GO terms were deemed significant if their p-value was less than or equal to 0.1.

## Results

In order to deeply understand the molecular differences between 402-91 and 402-91/ET cells, we performed multistep *omics* comparative analyses evaluating gene, miRNA and protein expressions.

### Gene expression data

A list of 2676 significant genes (corresponding to 3,083 non-unique probes) has been identified as differentially expressed between 402-91/ET and 402-91 cell lines (**Figure 1A**): 1473 (55%) underexpressed and 1203 (45%) overexpressed. Multiple probes in the microarray platform can be representative of a single gene; in our analysis more than 80% of probes belonging to the same gene behave concordantly. Only 505 genes represented by more than one probe in the platform have been identified as significant by a single probe. The complete list of differentially expressed probes is reported in **Table S1**. Remarkable is that a significant portion of the underexpressed (6%) and of the overexpressed (2%) genes are zinc finger proteins, i.e. proteins able to bind DNA (p = 0.0003 with hypergeometric distribution).

Functional enrichment has been performed separately for up and downregulated genes, both on GO categories (**Table 1**) and KEGG and MetaCore pathways (**Table 2**).

**Table 1:** Biological processes enrichment analysis within up and downregulated genes using Gene Ontology categories.

Category	Name	Adj p-value	Expression 402-91/ET vs 402-91
GO:0048522	positive regulation of cellular process	0,02	up
GO:0006915	apoptosis	0,03	up
GO:0008284	positive regulation of cell proliferation	0,04	up
GO:0031325	positive regulation of cellular metabolic process	0,04	up
GO:0051093	negative regulation of developmental process	0,05	up
GO:0001525	angiogenesis	0,05	up
GO:0001944	vasculature development	0,05	up
GO:0048523	negative regulation of cellular process	0,05	up
GO:0009893	positive regulation of metabolic process	0,05	up
GO:0001558	regulation of cell growth	0,05	up
GO:0010941	regulation of cell death	0,05	up
GO:0001501	skeletal system development	0,05	up
GO:0002684	positive regulation of immune system process	0,05	up
GO:0051249	regulation of lymphocyte activation	0,05	up
GO:0006954	inflammatory response	0,05	up
GO:0048513	organ development	0,05	up
GO:0043067	regulation of programmed cell death	0,06	up
GO:0050865	regulation of cell activation	0,06	up
GO:0051252	regulation of RNA metabolic process	0,0002	down
GO:0006350	transcription	0,0002	down
GO:0010468	regulation of gene expression	0,0006	down
GO:0080090	regulation of primary metabolic process	0,0007	down
GO:0060255	regulation of macromolecule metabolic process	0,0007	down
GO:0034645	cellular macromolecule biosynthetic process	0,0008	down
GO:0010556	regulation of macromolecule biosynthetic process	0,002	down
GO:0031326	regulation of cellular biosynthetic process	0,003	down
GO:0009889	regulation of biosynthetic process	0,003	down
GO:0051171	regulation of nitrogen compound metabolic process	0,003	down
GO:0031323	regulation of cellular metabolic process	0,003	down
GO:0019219	regulation of nucleobase. nucleoside. nucleotide and nucleic acid metabolic process	0,003	down
GO:0009887	organ morphogenesis	0,03	down
GO:0010563	negative regulation of phosphorus metabolic process	0,03	down
GO:0000082	G1/S transition of mitotic cell cycle	0,03	down
GO:0051329	interphase of mitotic cell cycle	0,05	down

**Table 2:** Pathways enrichment analysis within up and downregulated genes using KEGG and Metacore databases.

Database	Name	Adj p-value	Expression 402-91/ET vs 402-91
KEGG	NOD-like receptor signaling pathway	0,01	up
KEGG	Small cell lung cancer	0,03	up
KEGG	Vitamin B6 metabolism	0,05	up
KEGG	p53 signaling pathway	0,05	up
KEGG	PPAR signaling pathway	0,05	up
Metacore	Immune response_IL-17 signaling pathways	0	up
Metacore	Immune response_Alternative complement pathway	0	up
Metacore	Cytokine production by Th17 cells in CF	0,00021	up
Metacore	Cell cycle_Regulation of G1/S transition (part 1)	0,00023	up
Metacore	Cell cycle_Regulation of G1/S transition (part 2)	0,0006	up
Metacore	Development_TGF-beta-dependent induction of EMT via MAPK	0,0007	up
Metacore	Cytokine production by Th17 cells in CF (Mouse model)	0,00085	up
Metacore	Cell cycle_Nucleocytoplasmic transport of CDK/Cyclins	0,00122	up
Metacore	Development_TGF-beta-dependent induction of EMT via SMADs	0,00256	up
Metacore	Immune response_Th17-derived cytokines	0,00262	up
Metacore	Development_PEDF signaling	0,00361	up
Metacore	Cytoskeleton_Regulation of cytoskeleton rearrangement	0,00381	up
KEGG	Focal adhesion	0,001	down
KEGG	Notch signaling pathway	0,002	down
KEGG	Prion diseases	0,003	down
KEGG	ECM-receptor interaction	0,007	down
KEGG	Lysosome	0,01	down
KEGG	Gap junction	0,011	down
KEGG	Axon guidance	0,012	down
KEGG	Inositol phosphate metabolism	0,015	down
KEGG	Bladder cancer	0,03	down
KEGG	Tight junction	0,033	down
KEGG	Neurotrophin signaling pathway	0,034	down
KEGG	Insulin signaling pathway	0,035	down
KEGG	Valine, leucine and isoleucine degradation	0,038	down
KEGG	Chronic myeloid leukemia	0,044	down
KEGG	Endocytosis	0,044	down
KEGG	N-Glycan biosynthesis	0,047	down
KEGG	Adherens junction	0,051	down
Metacore	Cell adhesion_Amyloid proteins	0	down
Metacore	Immune response_Histamine signaling in dendritic cells	0,00001	down
Metacore	Signal transduction_cAMP signaling	0,00001	down
Metacore	Development_S1P2 and S1P3 receptors in cell proliferation and differentiation	0,00001	down
Metacore	Development_Blood vessel morphogenesis	0,00001	down
Metacore	Development_Notch Signaling Pathway	0,00002	down
Metacore	Development_A2A receptor signaling	0,00002	down
Metacore	Blood coagulation_GPCRs in platelet aggregation	0,00002	down
Metacore	Cell adhesion_Platelet aggregation	0,00002	down
Metacore	Cytoskeleton_Actin filaments	0,00002	down
Metacore	Development_Neurogenesis_Axonal guidance	0,00006	down
Metacore	Development_A2B receptor: action via G-protein alpha s	0,00008	down
Metacore	Signal transduction_Activation of PKC via G-Protein coupled receptor	0,00012	down
Metacore	Transcription_CREB pathway	0,00013	down
Metacore	PGE2 pathways in cancer	0,00018	down
Metacore	Signal Transduction_Cholecystokinin signaling	0,00052	down
Metacore	Cytoskeleton_Regulation of cytoskeleton rearrangement	0,00105	down
Metacore	Muscle contraction_Nitric oxide signaling in the cardiovascular system	0,00197	down
Metacore	Development_Hedgehog signaling	0,00287	down
Metacore	Cell adhesion_Leucocytechemotaxis	0,00747	down

Most of the genes overexpressed in 402-91/ET cell line are implicated in apoptosis, regulation of cell proliferation, angiogenesis, vascular development and negative regulation of developmental processes (enclosed the adipocyte differentiation category). On the other hand, pathways related to metabolism, gene expression and cell cycle progression are mainly populated by downregulated genes. Pathways associated to cancer (p53 signaling pathway and small cell lung cancer) are involved in trabectedin resistance as well as immunoregulatory mechanisms like PPARs and NOD-like receptors signaling. Some key genes involved in cell cycle and regulation of apoptosis have been validated with qRT-PCR (see **Table S2** and section below).

### miRNA expression data

Inferential analysis identified a list of 47 miRNAs that showed an altered regulation between 402-91/ET and 402-91 cell lines (**Figure 1B**): 23 overexpressed and 24 underexpressed (see **Table S3**). Among these, we found two miRNA clusters (defined as miRNAs whose chromosomal position is less than 1000 kb) on chromosome 13 (miR-17*, miR-19a, miR-19b, miR-20a, miR-92a) and 19 (miR-99b, let-7e, miR-125a-5p) and the miRNA families miR-320, miR-19 and miR-29.

Moreover, we performed a functional enrichment analysis on putative targets of significantly altered miRNAs in 402-91/ET cell line (see **Table 3**). The *in silico* predicted targets were cleaned out of those genes without differential expression. Gene targets of miRNAs regulation belong to categories involved in immune response, regulation of cell death, apoptosis and anti-apoptosis and G1/S transition of mitotic cell cycle. Among these genes, there were several known oncogenes playing a key role in tumor progression in melanoma, bladder and lung cancer. Some key miRNAs involved in cell cycle and regulation of apoptosis have been validated with qRT-PCR (see **Table S2** and section below).

**Table 3:** GO biological processes and KEGG and Metacore pathway enrichment analysis within miRNA targets.

Category	Name	Adj p-value
**Gene Ontology**		
GO:0001525	angiogenesis	0,01
GO:0000082	G1/S transition of mitotic cell cycle	0,01
GO:0043067	regulation of programmed cell death	0,02
GO:0051252	regulation of RNA metabolic process	0,02
GO:0042981	regulation of apoptosis	0,02
GO:0006355	regulation of transcription. DNA-dependent	0,02
GO:0009887	organ morphogenesis	0,02
GO:0001944	vasculature development	0,03
GO:0006916	anti-apoptosis	0,03
GO:0048705	skeletal system morphogenesis	0,03
GO:0001568	blood vessel development	0,04
GO:0046395	carboxylic acid catabolic process	0,07
GO:0048514	blood vessel morphogenesis	0,07
GO:0016477	cell migration	0,09
**Pathway**		
Metacore	PGE2 pathways in cancer	0
Metacore	Immune response_Histamine signaling in dendritic cells	0,00013
Metacore	Signal transduction_cAMP signaling	0,00015
Metacore	Development_S1P1 receptor signaling via beta-arrestin	0,00019
Metacore	Muscle contraction_Regulation of eNOS activity in endothelial cells	0,00021
Metacore	Immune response_Histamine H1 receptor signaling in immune response	0,00022
Metacore	Cell adhesion_Chemokines and adhesion	0,00022
Metacore	Development_Mu-type opioid receptor signaling via Beta-arrestin	0,00032
Metacore	Immune response_PGE2 signaling in immune response	0,00038
Metacore	Development_S1P2 and S1P3 receptors in cell proliferation and differentiation	0,00055
KEGG	ECM-receptor interaction	0,009

### Signature validation

**Figure 2:**
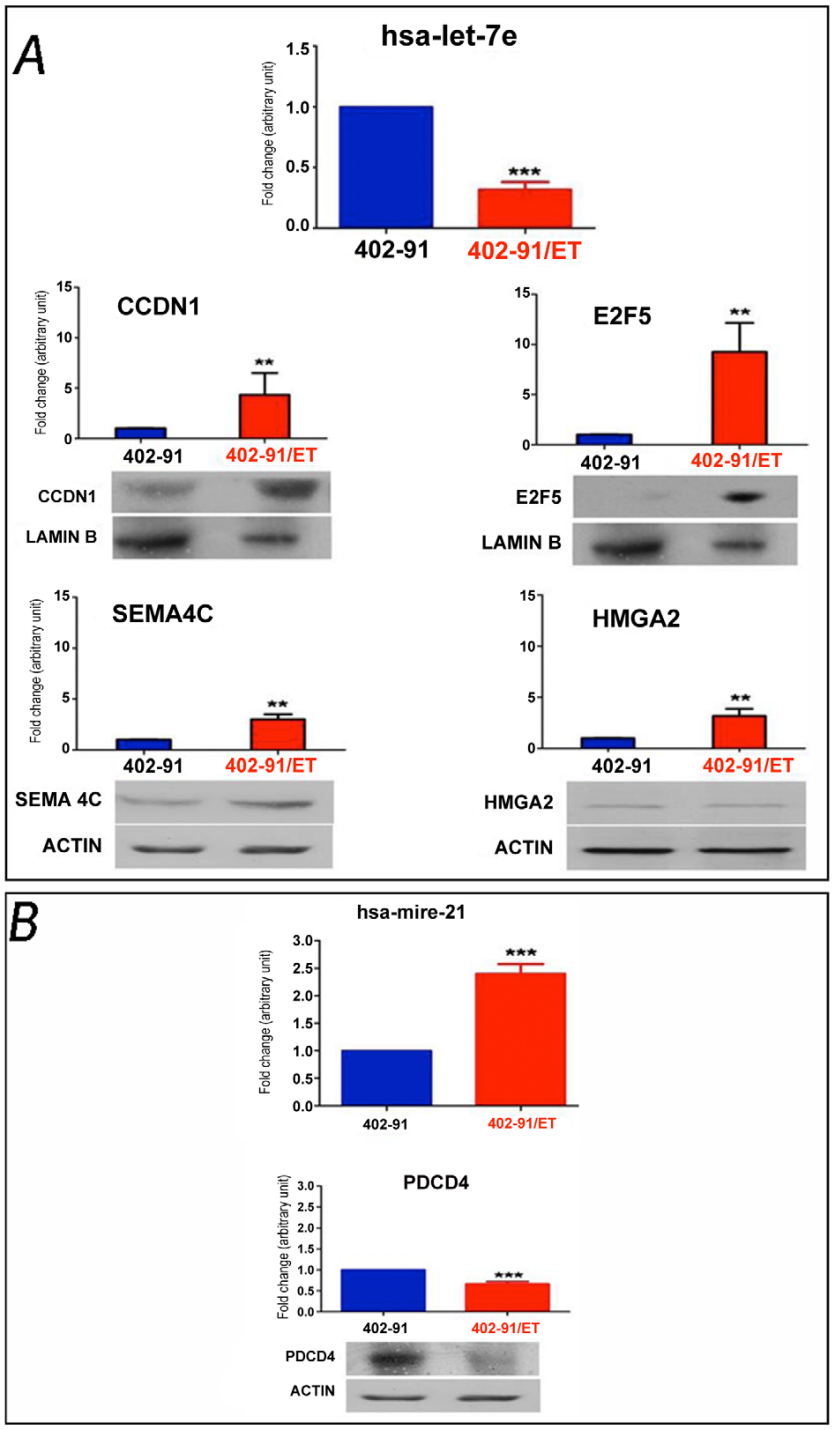
Signature validation of miRNA-mRNA and proteins found differentially expressed between 402-91/ET and 402-91 cell line. **Panel A**. qRT-PCR and Western blot analysis showing differences in the expression levels of let-7e and its downstream targets (CCDN1, E2F5, SEMA4C, HMGA1 and HMGA2). **Panel B**. qRT-PCR and Western blot for miR-21 and its downstream target, PDCD4. qRT-PCR data are the mean of three independent experiments performed in triplicate and calculated with the 2^−ΔΔCt^ method as described in the material and method section. The control 402-91 cells values are arbitrarily set as 1. Bars are +/− SD. * is significant with p<0.05, ** p<0.01, ***p<0.001, as assessed with Student T-test. Western blot is representative of at least two independent experiments.

Apoptosis and cell cycle seem to be the most involved pathways in trabectedin resistance in 402-91/ET cell line either on transcriptional or on translational levels. Thus, to support the robustness of our analysis, differences in expression of genes and miRNA, belonging to the apoptosis and cell cycle pathways were validate in an independent batch of 402-91 and 402-91/ET cells. Representative results for let-7e and miR-21 are reported in **Figure 2**. As expected, let-7e resulted three folds downregulated (p<0.001) and miR-21 two folds upregulated (p<0.0001) in 402-91/ET compared to 402-91cells. qRT-PCR analysis of let-7e downstream targets confirmed four folds upregulation of CCDN1 (p<0.01), three folds upregulation for SEMA4C (p<0.01) and nine folds upregulation for E2F5 (p<0.001) in the resistant compared to the sensitive cell line (**Figure 2A**). Western blot analysis confirmed at protein level such differences. Analysis of PDCD4, a downstream target of miR-21, was confirmed to be downregulated in the resistant cells (1.5 times, p<0.0001) at both mRNA and protein levels (**Figure 2B**). These results confirmed our *omics* analysis even if the effect of let-7e on HMGA2 needs to be discussed separately. The three folds upregulation of HMGA2 at the mRNA level in 402-91/ET cells (p<0.01) was not further confirmed by western blot analysis (**Figure 2A**), suggesting that other mechanisms, not directly related to control of mRNA stability by miR-21, are involved in the HMGA2 protein stability.

### Antibody array data

**Figure 3:**
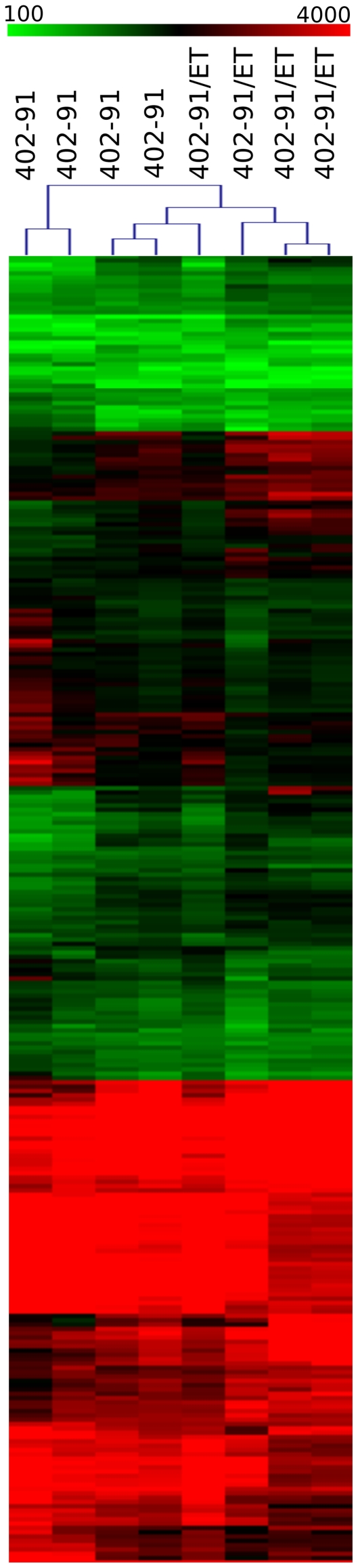
Heat map and cluster analysis of the 336 differentially expressed proteins between resistant and sensitive cell lines. Red and green represent respectively differentially upregulated and downregulated proteins in 402-91/ET cell lines.

Protein profiling identified 336 proteins that were significantly (FDR<0.1) different between sensitive and resistant cell lines (**Table S4**): 148 upregulated and 188 downregulated (see **Figure 3**). Cluster dendrograms of Figure 1A, 1B and 3 were performed on different data sources (genes, miRNAs and proteins expression levels) characterised by different sizes, means and range of values. Since hierarchical clusteringis highly sensitive to data structure, some differences in dendrograms are expected, given the intrinsic characteristics of the data. According to the experimental design, gene, miRNA and protein expression profiles were quantified using three different biological replicates, while within each batch technical replicates were performed. Thus, the difference in the dendrogram structures is due to the combination of these effects: biological and intrinsic data differences.

In order to assess the biological meaning of the differences in the protein expression, we performed enrichment analysis on GO terms, cellular networks and pathway maps using DAVID web tool and MetaCore software.

MetaCore analysis showed a prevalence of apoptosis-survival related functions for the upregulated proteins: the top three categories from enrichment analysis on GO terms showed prevalence of terms associated to cell death (p<0.0001). Of the top three pathways, two were associated with apoptosis (FAS signaling cascades and TNF family pathways), and “death domain receptors and caspases in apoptosis” was identified as significantly enriched in process networks. On the other hand, GO terms over-represented in downregulated proteins mainly involved cellular component reorganization and affected pathways and networks including AKT signaling, cytoskeleton remodeling and G1/S cell cycle regulation (**Table 4**). Other involved functions were related to cell cycle, cytoskeletal remodeling and growth factor regulation.

**Table 4:** Gene Ontology categories and KEGG, PANTHER and Metacore pathway enrichment analysis within up and down regulated proteins.

Category	Name	p-value	Expression 402-91/ET vs 402-91
**GeneOntology**			
GO	apoptosis	0,00000	up
GO	programmedcelldeath	0,00000	up
GO	celldeath	0,00000	up
GO	cellular component organization	0,00000	down
GO	cellular component organization or biogenesis	0,00000	down
GO	cellular component organization at cellular level	0,00000	down
**Pathway**			
KEGG	hsa04115:p53 signalingpathway	0,00027	up
KEGG	hsa04210:Apoptosis	0,00140	up
KEGG	hsa05219:Bladder cancer	0,00397	up
KEGG	hsa05218:Melanoma	0,04945	up
KEGG	hsa05212:Pancreatic cancer	0,05277	up
KEGG	hsa04010:MAPK signalingpathway	0,07292	up
KEGG	hsa05200:Pathways in cancer	0,07840	up
KEGG	hsa04650:Natural killer cell mediated cytotoxicity	0,14232	up
PANTHER	P00006:Apoptosis signalingpathway	0,00052	up
PANTHER	P00059:p53 pathway	0,01579	up
Metacore	Apoptosis and survival_p53-dependent apoptosis	0	up
Metacore	Apoptosis_Death Domain receptors &caspases in apoptosis	0	up
Metacore	Cell adhesion_Amyloid proteins	0	up
Metacore	Cell cycle_G1-S Growth factor regulation	0,00001	up
Metacore	Apoptosis and survival_Role of IAP-proteins in apoptosis	0,00026	up
Metacore	Translation _Regulation of EIF2 activity	0,00084	up
KEGG	hsa04110:Cell cycle	0,00000	down
KEGG	hsa05200:Pathways in cancer	0,00235	down
KEGG	hsa04510:Focal adhesion	0,00681	down
KEGG	hsa04210:Apoptosis	0,01537	down
KEGG	hsa05212:Pancreatic cancer	0,03870	down
PANTHER	P00006:Apoptosis signalingpathway	0,00022	down
PANTHER	P00020:FAS signalingpathway	0,00130	down
Metacore	Cytoskeleton remodeling_TGF, WNT and cytoskeletal remodeling	0,00000	down
Metacore	Signaltransduction_AKTsignaling	0,00000	down
Metacore	Cytoskeletonremodeling_Cytoskeletonremodeling	0,00000	down
Metacore	Cell cycle_G1-S Growth factor regulation	0,00000	down
Metacore	Cell cycle_G1-S Interleukin regulation	0,00001	down
Metacore	Cell cycle_Core	0,00015	down

Likewise, the analysis performed with DAVID identified among the highest significant results pathways related to apoptosis, stress induction of HSP and the p53 pathway for upregulated proteins, and cell cycle, AKT signaling, and FAS signaling for downregulated proteins (**Table 4**).

### CHOP transcription factor binding site analysis

It has been reported that FUS-CHOP is the main molecular target of trabectedin in myxoid liposarcoma cell line [12], [13]. Under the assumption that the FUS-CHOP binding site is the same of CHOP (then of the heterodimer CHOP-C/EBPá), we tried to identify putative miRNAs transcriptionally regulated by the chimera and therefore having an enriched CHOP motif in their promoter regions.

Transcription factor binding sites enrichment analysis performed on separately up or downregulated genes (oPOSSUM web tool with Z-score>5 and p-value<0.5) identified respectively 10 and 11 binding sites enriched (see **Table S5**). CHOP binding site was not identified as enriched.

The identification of miRNA promoter regions is still a challenging task, since putative miRNA regulatory regions could be several thousands of bp upstream of the pri-miR. There are only a limited number of experimentally validated miRNA promoters. In particular only few of the differentially expressed miRNAs have an experimentally validated promoter region. In this context, TFBS enrichment analysis on the promoter regions of the differentially expressed miRNAs would be misleading. However, we could identify miRNAs among those differentially expressed with an experimentally validated promoter with a putative CHOP binding site. Using mirGen2.0 web tool, we found that miR-130a (downregulated in 402-91/ET cell line) and miR-21 (upregulated in 402-91/ET cell line) have a CHOP-C/EBPá binding site in their experimentally validated promoter regions, while using CircuiDB database [29] we found that miR-7 (upregulated in 402-91/ET cell line) has a CHOP-C/EBPá binding site in its computationally identified promoter region.

### Integrative analysis and network reconstruction

#### Integration of miRNA and mRNA expression data

miRNA effect on mRNA degradation can be detected by the anti-correlation relationship between miRNA and gene expression. To reconstruct miRNA-mRNA network in this section we focused only on miRNAs having an inverse relationship with their respective mRNA targets.

Using only the list of differentially expressed miRNAs and genes we reconstructed the network in which nodes represent both genes and miRNAs (colored according to their expression levels) while edges represent computationally identified miRNA-target interactions. The entire resulting network is available in **Figure S1 and Table S6**.

**Figure 4:**
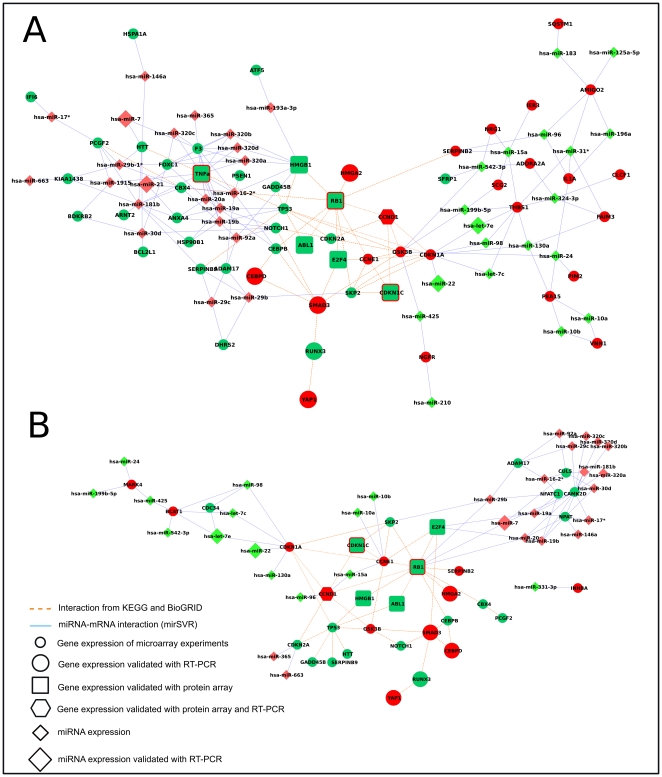
Post-transcriptional network. miRNA and mRNA subnetworks representing negative regulation of apoptosis (Panel A) and cell cycle (Panel B). Small circle: differentially expressed genes found by microarray analysis. Big circle: differentially expressed genes validated by qRT-PCR. Square: differentially expressed genes encoding a protein found differentially expressed using protein array. Exagon: differentially expressed genes validated with qRT-PCR encoding a protein found differentially expressed using protein array. Small diamonds: differentially expressed miRNAs found by array analysis. Big diamonds: differentially expressed miRNAs validated by RT-PCR. Filled colors inside represent gene expression, border colors outside represent protein level.

**Figure 5:**
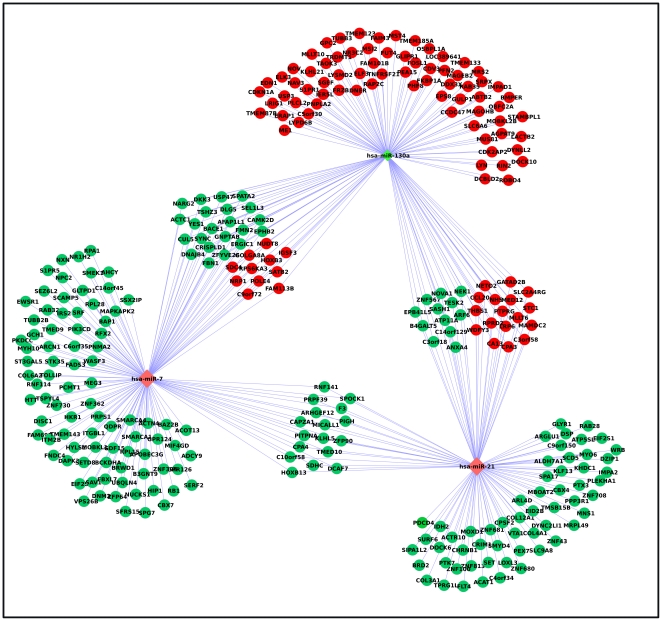
Target genes shared by miR-7, miR-21 and miR-130a. Nodes represent miRNAs and mRNA gene expression. Red represents gene/miRNA upregulation, green downregulation.

We then focused on specific network modules (sub-networks), corresponding to pathways or specific miRNAs found to be interesting according to the biological problem under study (**Figure 4**). In particular we selected genes involved in negative regulation of apoptosis (**Figure 4A**) and cell cycle (**Figure 4B**). Then we selected the putative targets of those miRNAs (miR-7, miR-21 and miR-130a) with experimentally validated or computationally identified CHOP- C/EBPá binding sites in their promoter regions (**Figure 5**). Several miRNAs and genes identified as differentially expressed have been validated in qRT-PCR as previously reported (**Figure 2** **; Table S2**).

#### Integration of miRNA-protein expression levels

**Figure 6:**
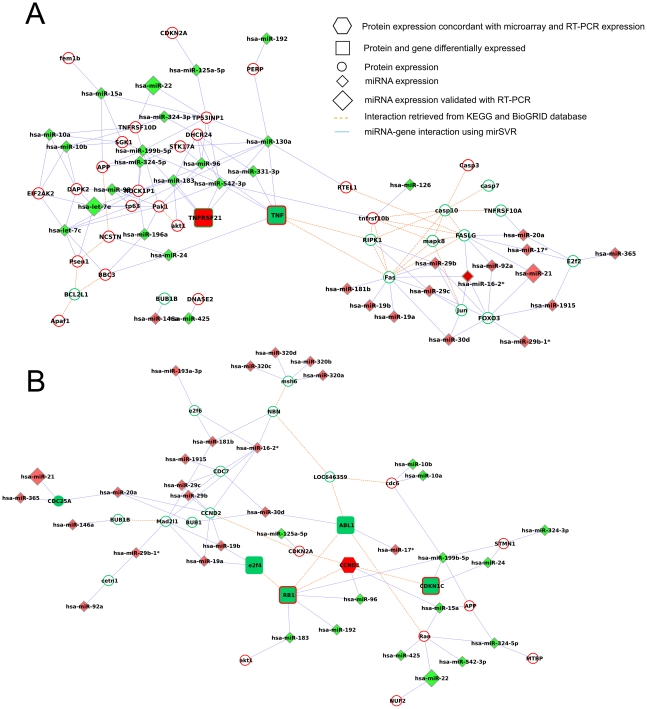
Pre-translational network. miRNA and protein subnetworks representing apoptosis (Panel A) and cell cycle (Panel B). Small circle: differentially expressed genes found by microarray analysis. Square: differentially expressed genes encoding a protein found differentially expressed using protein array. Exagon: differentially expressed genes validated with qRT-PCRencoding a protein found differentially expressed using protein array. Small diamonds: differentially expressed miRNAs found by array analysis. Big diamonds: differentially expressed miRNAs validated by qRT-PCR. Filled colors inside represent gene expression, border colors outside represent protein level.

As the effect of miRNAs does not limit to mRNA degradation but also translational repression, we integrated miRNA and protein expression using the approach described previously (see above). As apoptosis and cell cycle were mainly involved in trabectedin resistance as reported in the previous enrichment results (**see** **Table 4**), we generated a miRNA-protein network for these two pathways (**Figure 6** **, panels A and B respectively**).

#### Integration of mRNA and protein levels

The simultaneous analysis of differentially expressed genes and proteins showed a core of 22 genes for which not only the mRNA expression but also the protein levels were significantly altered between 402-91/ET and 402-91 cell lines. Within this core it is possible to clearly identify two sets of genes: 54% of genes with mRNA positively correlated with protein levels (r = 0.96) and 45% of genes with mRNA negatively correlated with protein levels (r = −0.6).

This result can be easily explained considering two points: i. mRNA and protein levels are not quantified from exactly the same biological sample but rather from two different biological replicates ii. mRNAs and proteins have different turnover.

#### Integration of miRNA-mRNA-protein expression levels

miRNA, mRNA and protein can be combined in modules in which a miRNA acts on messenger targets and the resultant effect is measured on the protein.

**Figure 7:**
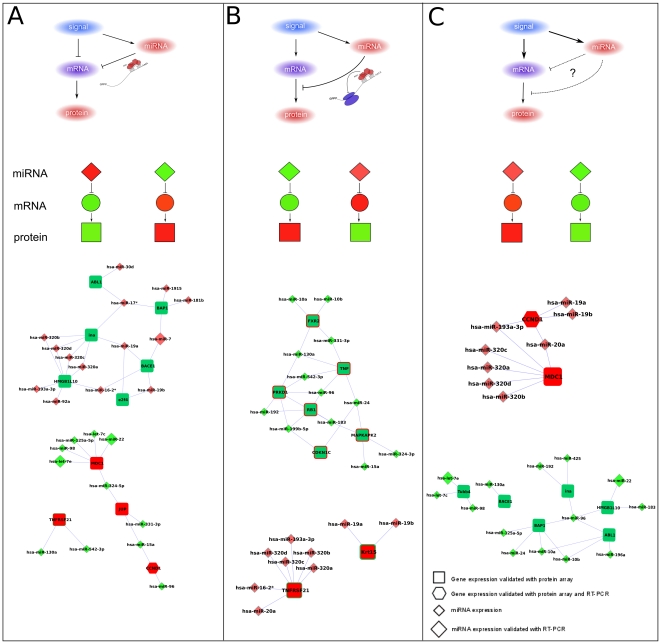
Regulatory loops identified through the combination of miRNA, mRNA and protein expression levels. **Panel A**. Coherent regulatory loops: up/down miRNA regulating the target mRNA through degradation. **Panel B**. Incoherent regulatory loops: an up/down miRNA regulating the protein level through translational repression. **Panel C**. Incoherent regulatory loops: an up/down miRNA whose effect is not sufficient to overcome the effect of another external signal and thus it is impossible to hypothesize if miRNA acts as mRNA degradation or translational repressor.

In our study the combination of post-transcriptional and pre-translational regulatory networks allows the identification of triplets (miRNA-mRNA-protein) representing different miRNA action scenarios. Mapping the levels of expression on these modules allows us to identify putative different types of regulatory loops [32]. The possible loops to be considered regard **i)** an up/down miRNA regulating the target mRNA through degradation (type A, **Figure 7A**), **ii)** an up/down miRNA regulating the protein level through translational repression (type B, **Figure 7B**), **iii)** an up/down miRNA whose expression does not support an action on the predicted targets or an up/down miRNA whose effect is not sufficient to overcome the effect of another external signal (and thus is impossible to hypothesize if miRNA acts as mRNA degradation or translational repressor) (type C, **Figure 7C**).

Depending on the signal, loops can be divided into two classes: coherent (loop of type A; characterized by mutually exclusive expression between miRNA and its targets) and incoherent (loops of type B and C; characterized by coexpression of miRNA and its target) [33]. In the coherent loops both pathways from the signal to the target have the same effect (both repressing or activating target expression), while in the incoherent ones the two pathways have opposite effects [33]. From a biological point of view, incoherent loops of type B have an easy interpretation while incoherent loops of type C have a less clear explanation and then usually require further investigations. **Figure 7** shows in details these possibilities jointly with the loops found in our data. In particular we found 38 putative loops of type A, 34 of type B and 28 of type C. Looking at the coherent loops, we identified 3 loops involving differentially expressed miRNAs with roles in drug resistance and/or potentially regulated by FUS-CHOP chimera (let-7e, miR-130a, miR-98 and miR-7). These loops have as potential targets MDC1, TNFRSF21 (overexpressed in 402-91/ET), BACE1 and BAP1 (underexpressed in 402-91/ET). MDC1 activates the intra-S phase and G2/M phase cell cycle checkpoints, in response to DNA damage. It has been demonstrated that MDC1 is implicated in cellular apoptotic response [33]. TNFRSF21 encodes a protein that is a member of the TNF-receptor superfamily. This receptor has been shown to activate NF-kappaB and MAPK8/JNK and induce cell apoptosis. The protein encoded by BAP1 localizes to the nucleus and it interacts with the RING finger domain of the breast cancer 1, early onset protein (BRCA1). This gene is thought to be a tumor suppressor gene that functions in the *DNA* repair. As far as the incoherent circuits of type B concerns we identified two loops with miR-130a that have PRKD1 and TNF as potential targets and one loop regarding a cluster of miRNAs (miR-320) with TNFRSF21. On the other hand, as far as circuits of type C concerns we identified 3 loops with let-7e and miR-98, FXR2, TUBB4 genes and maintenance of genomic stability pathway.

## Discussion

Despite its complex molecular heterogeneity, cancer results from the integration of at least ten common pathways that drive tumor progression and therapy response [34]. It is now becoming clear that pathway integration rather than single gene manipulation could represent the basis to understand malignant transformation and therapy response.

Based on this hypothesis, in the current study we exploited different transcriptomic (miRNA and GE array) and proteomic (protein array) platforms to generate, in a well defined *in vitro* model of tumor cells, an integration map of the key pathways possibly involved in the mechanism of resistance to trabectedin.

In this perspective, our study is one of the first attempts towards the combination of the three layers of gene regulation (transcriptional, post-transcriptional and translational) for the identification of those pathways and networks playing a critical role in trabectedin action.

In particular, in this work, we looked for changes in molecular behavior due to an intensive and prolonged treatment with trabectedin that was performed to induce drug resistance. Then, using a systems biology approach we systematically screened the possible sources of variability in mRNAs, miRNAs and protein expressions. Our integration strategy started by extracting individual results from the three different layers and then combining them to increase their power.

### Gene expression data

Regarding gene expression data, a significant portion of the underexpressed (6%) and overexpressed (2%) genes are zinc finger (ZF) proteins. ZF proteins are multiple and play important role in the metabolism of normal cells. There are many reports on the inhibition of ZF transcription factors in early as well as advanced stages of oncogenesis, including the impairment of signal transduction [35]. These results are also in agreement with the recent findings of Duan et al. [36] who performed gene expression analysis on chondrosarcoma cell lines resistant to trabectedin. Remarkably, the authors found some ZF proteins to be differentially expressed and suggest that ZF proteins are involved in the mechanism of trabectedin resistance. However, differently from Duan et al. [36] we did not find overexpression of ZNF93 and ZNF43 in the resistant 402-91/ET cell line.

Interestingly, we found BIRC2 (which encodes for c-IAP1 protein) overexpressed in 402-91/ET cells. c-IAP1 inhibits apoptosis by binding to TNF receptor-associated factors TRAF1 (that we found overexpressed in resistant cells) and TRAF2. It has recently been reported that BIRC2 is associated with resistance of esophageal squamous cell carcinomas to drug-induced apoptosis [37] and that c-IAP1 could be a novel predictive marker for resistance to radiotherapy in cervical squamous cell carcinomas [37].

The 402-91/ET cell line seems to be characterized by a reduced apoptosis and an increased cell proliferation. It is noteworthy that pathways involved in cell adhesion are enriched in downregulated genes, suggesting a loss of 402-91/ET intracellular communications and altered cellular processes such as proliferation, migration and differentiation.

### miRNA expression data

miRNAs are involved in processes such as development, carcinogenesis, cell survival, and apoptosis, as well as cellular sensitivity to anticancer drugs [38]. However, opposite effects can be seen towards the same compound in different tumor types, suggesting a complex relationship between miRNAs and drug resistance [39].

Among differentially expressed miRNAs in 402-91/ET we found miR-21, let-7e, miR-192, miR-130a and miR-98, whose ability to affect the potencies of a number of anticancer agents have been recently reported. Several studies recently reported a relation between the overexpression of miR-21 and the resistance to a variety of anticancer drugs. In particular, Shi and colleagues [40] demonstrated a role of miR-21 in temozolomide resistance in glioblastoma cells. Moreover, the overexpression of miR-21 has also been found to induce resistance against cytosine arabinoside (Ara-C) and arsenic trioxide. Targeting of PDCD4 (underexpressed in 402-91/ET cells) by miR-21 prevented Ara-C-induced apoptosis in the leukemic HL60 cell line [41], whereas in several myelogenous cell lines reduced expression of PDCD4 by miR-21 targeting prevented arsenic trioxide-induced apoptosis [42].

Let-7e, and miR-130a were found underexpressed in a panel of paclitaxel- and cisplatin-resistant cells lines [43]. Furthermore, Herbert et al. [44] predicted HGMA2 (overexpressed in microarray and validated with qRT-PCR) as a target for miR-98, and showed their involvement in promotion of resistance to doxorubicin and cisplatin, while Boni and colleagues suggested a role of miR-192 in 5-fluorouracil resistance in colorectal cancer [45].

In general, pathway analysis on microRNA target genes highlights a subset of the pathways previously found in gene expression analysis. This means that in some specific pathways both levels of transcription are co-involved in the development of resistance to trabectedin. Cell cycle, angiogenesis, regulation of transcription and apoptosis-survival are the mostly over-represented pathways.

### CHOP transcription factor binding site

We investigated the presence of differentially expressed miRNAs, potentially transcriptionally regulated by FUS-CHOP chimera with an enriched CHOP motif in their promoter regions. We found three differentially expressed miRNAs (miR-21, miR-130a, miR-7) having either computationally or experimentally validated CHOP binding sites. Remarkably two of them, miR-21 and miR-130a, were previously discussed because of their documented involvement in drug resistance.

Among our differentially expressed genes we found 149 putative targets of miR-7 (with an anti-correlated expression profile), which are mainly involved in cell cycle, chromatin and cytoskeleton organization. Among them we found PIK3CD, RB1, CAMK2D, CUL5. There are emerging evidences about the involvement of PI3K signaling cascade in myxoid liposarcoma. The overexpression of receptor tyrosine kinases MET, IGF1R and IGF2 promote cell survival through both the PI3K/Akt and the Ras-Raf-ERK/MAPK pathways [46].

Mir-21 had 99 putative anti-correlated targets mostly involved in the regulation of transcription (CBX4, KLF13, PDCD4 and several ZF proteins).

Among the 118 anti-correlated putative targets of miR-130a, we identified genes involved in vasculature development (ROBO4, SIPR1, NRP1, ELK3, EDN1, THBS1) and cell motion, in particular FOSL1, PLCL2 and LYN. FOSL1 dimerizes with proteins of the JUN family (JUNB is overexpressed in resistant cell line) forming the transcription factor complex AP-1; FOS proteins have been implicated as regulators of cell proliferation, differentiation, and transformation. Furthermore, LYN has been recently shown to act as a mediator of tumor invasion.

### Antibody array data

Gene and miRNA expression profiles are able to separate 402-91/ET from 402-91 cell lines (**Figure 1A and 1B**), while protein expression dendrogram (**Figure 3**) has a more heterogeneous behaviour. 402-91 cell line is divided into two groups; one 402-91/ET sample clusters with 402-91 ones. It is much more difficult to quantify protein expression than gene expression in a multiplex manner than for gene expression, due to the larger variability in the physico-chemical properties of proteins [**31**]. Therefore, this discrepancy could be due to the higher variability of protein arrays than that of mRNA and miRNA assessment.

GO enrichment analysis on differentially expressed proteins seems to be much more focused than the same analysis on genes. In particular, apoptosis-survival and cell cycle are categories capable of collecting the greatest number of differences between sensitive and resistant cells. Among the over-represented pathways we found p53, apoptosis and MAPK.

In particular we found CCND1, CCND2, BBC3, CDKN2A, PERP involved in p53 signaling, RB1, E2F2, E2F3, E2F4, SMAD4, CDC14, CDC25A, CDC6, CDC7 involved in cell cycle. Some of these proteins are involved in the regulation of transcription in G1/S phase of the mitotic cell cycle. G1/S transition is a rate-limiting step in cell cycle progression [47].

The cyclin D1 proto-oncogene is a key regulator of G1 to S phase progression in several cell types. Together with CDK4 and CDK6, cyclin D1 forms active complexes that promote cell cycle progression by phosphorylating and inactivating the retinoblastoma protein (RB1). RB1 in turn leads to the release of the E2F family of transcription factors driving the expression of several genes associated with S phase progression [48]. A number of therapeutic agents have been observed to induce cyclin D1 degradation *in vitro*, indicating that such an induction may offer a useful avenue for therapeutic intervention.

### Integrative analysis and network reconstruction

After the analysis and the discussion of each regulatory aspect (mRNAs, miRNAs and proteins) separately, we combined all the biological evidences within a systems biology approach in order to have a wider perspective of the regulatory phenomena acting during trabectedin resistance.

miRNAs act as post-transcriptional regulators of gene expression. The reduction of protein levels is the final result of the regulation that can be achieved through two strategies: (i) mRNA degradation or (ii) translational repression. The effects of the two modes of action can be evaluated at different points of the transcription-translation pathway. Focusing on the two main highly correlated pathways that seem to be involved in trabectedin resistance (negative regulation of apoptosis and cell cycle), we reconstructed two different networks: i) the network composed of differentially expressed mRNAs targeted by differentially expressed miRNAs that allow us to study the regulation at the transcriptional level, hereafter the “post-transcriptional regulatory network” and ii) the network composed by differentially expressed miRNAs leading to differentially expressed proteins, hereafter “pre-translational regulatory network”.

It is clear the central role that CCND1, RB1, E2F4, TNF, CDKN1C, ABL1, TNFRSF21 play in both post-transcriptional and pre-translational regulatory networks. In particular, they act as a bridge between the two parts of the network with opposite regulations: upregulated genes/protein and downregulated miRNA and downregulated genes/protein and upregulated miRNA.

In some cases the involvement of miRNAs can explain the discordant relation between the transcriptome and the proteome. In a seminal review Inui et al. [32] describe the role of miRNAs in pathways as decision maker elements to discriminate real versus too weak or too transient signals. In this study we identified 3 loops involving differentially expressed miRNAs with roles in drug resistance; these loops have as potential targets MDC1 (overexpressed in 402-91/ET), BACE1 and BAP1 (underexpressed in 402-91/ET).

### Concluding remarks

The present study is one of the first works that try to screen the behavior of genes, microRNAs (miRNAs) and proteins in order to reconstruct *in silico* the regulatory networks leading to resistance to anticancer agents such as trabectedin. Diverse sources of *omic* data have been used to describe altered regulatory mechanisms within a transcriptional, post-transcriptional and translational perspective. The combination of the three layers of regulation gave us the possibility to reconstruct putative gene-miRNA-protein circuits altered in trabectedin resistance. We found that transcriptome and proteome data agreed in recognizing anti-apoptosis and cell cycle proliferation as the two main biological processes characterizing the difference between 402-91 and 402-91/ET cell lines. All the hallmarks of cancer seem to be altered in the resistant cell line, raising the hypothesis that the mechanism of resistance is acting with more strength on the same molecular markers triggered by cancer development.

On the other hand, post-transcriptional analysis revealed two miRNAs (miR-21 and miR-130a) already identified as playing a role in drug resistance and putatively regulated by the FUS-CHOP chimera, which could be good markers for future functional studies. Most of miR-21 and miR-130a targets are known oncogenes or oncosuppressors highlighting their involvement in malignancies.

Once validated on *in vivo* models this approach might allow the identification of druggable targets for trabectedin-resistant malignancies.

## Supporting Information

Figure S1 miRNA and mRNA network reconstruction using mirSVR as target prediction tool. Colors represent expression (green and red for under and overexpressed genes, light green and light red for under and overexpressed miRNA) and edges between miRNA and gene represent mirSVR predictions, while gene-gene edges represent validated gene-gene interactions from KEGG database.
(PDF)

Table S1 Complete list of probes identifying differentially expressed genes.
(XLSX)

Table S2 Complete list of validated mRNA and miRNA expression levels using qRT-PCR.
(PDF)

Table S3 Complete list of differentially expressed miRNAs.
(PDF)

Table S4 Complete list of differentially expressed proteins.
(XLSX)

Table S5 Complete list of predicted transcription factor binding sites enriched in the promoter region of up and downregulated gene in 402-91/ET cell lines.
(PDF)

Table S6 Simple interactions format (sif) file for gene-gene and miRNA-gene interactions of the whole network reported in Figure S1.
(XLSX)
