# Supplementary figures and images for: A Systems Biology Approach to Characterize the Regulatory Networks Leading to Trabectedin Resistance in an In Vitro Model of Myxoid Liposarcoma
Source: PLoS One. 2012 Apr 16;7(4):e35423. doi: 10.1371/journal.pone.0035423 (PMC3327679; doi:10.1371/journal.pone.0035423)

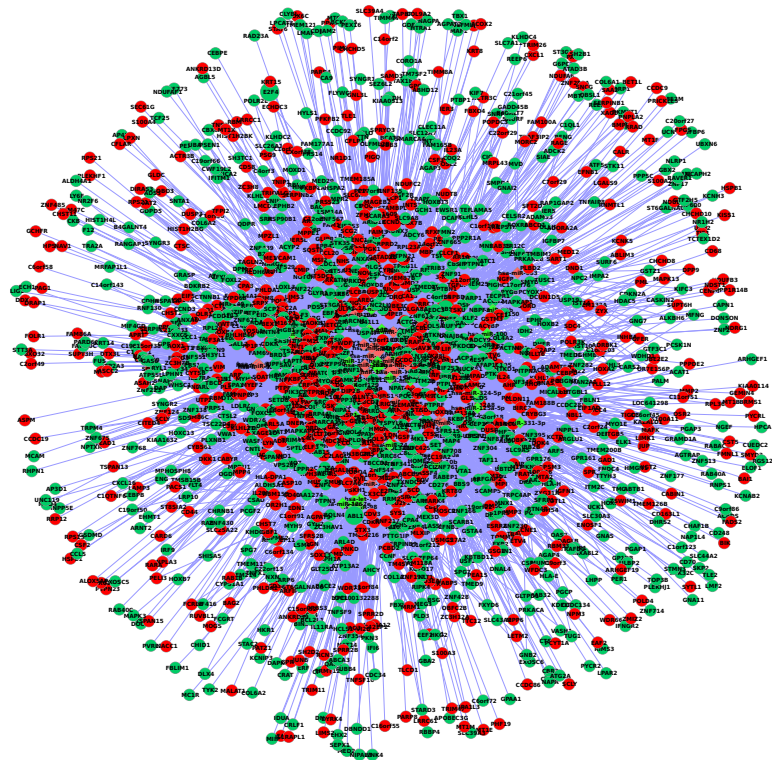

Supplement: Figure S1 — miRNA and mRNA network reconstruction using mirSVR as target prediction tool. Colors represent expression (green and red for under and overexpressed genes, light green and light red for under and overexpressed miRNA) and edges between miRNA and gene represent mirSVR predictions, while gene-gene edges represent validated gene-gene interactions from KEGG database. (PDF) [file pone.0035423.s001.pdf]
