# Supplementary material for: A Systems Biology Approach to Characterize the Regulatory Networks Leading to Trabectedin Resistance in an In Vitro Model of Myxoid Liposarcoma
Source: PLoS One. 2012 Apr 16;7(4):e35423. doi: 10.1371/journal.pone.0035423 (PMC3327679; doi:10.1371/journal.pone.0035423)
Supplement: Table S2 — Complete list of validated mRNA and miRNA expression levels using qRT-PCR. (PDF) [file pone.0035423.s003.pdf]

Expression 402-91/ET vs 402-91			
**gene symbol**	**Avg Expression**	**SD (+/-)**	**microarray expression**
c/EBPD	3.4	0.06	3.73
c/EBPE	0.33	0.07	0.27
FOSL 1	6.8	2.3	8.00
FOSL 2	6.8	2.3	2.46
GSTM3	4.19	0.38	4.00
HMGA2	3.18	0.7	6.96
EGR	0.31	0.6	0.29
SMAD3	2.25	0.25	3.66
IAP3	55.3	11.28	36.76
YAP	6.44	0.98	3.73
IAP2	6.5	0.37	5.28
RUNX	0.32	0.024	0.16
PTX3	**0.2**	0.06	0.27
BIRC3	138	47	36.76
YAp1	8.4	0.18	7.46
BIRC2	14	1.6	5.28
DAP	0.3	0.04	0.16
TNFRSF25	0.24	0.02	0.29
IGFBP3	3	0.04	6.06
FN1	0.26	0.03	0.50
CCND1	4.3	2	3.73
E2F5	9.25	2.9	3.73
SEMA4C	3	0.5	2.46
let-7e	0.32	0.06	0.33
miR-22	0.6	0.05	0.46
miR-146a	6.6	1.1	9.00
miR-21	2.4	0.17	1.89
miR-7	3.90	1.30	2.31
