# Supplementary material for: A Systems Biology Approach to Characterize the Regulatory Networks Leading to Trabectedin Resistance in an In Vitro Model of Myxoid Liposarcoma
Source: PLoS One. 2012 Apr 16;7(4):e35423. doi: 10.1371/journal.pone.0035423 (PMC3327679; doi:10.1371/journal.pone.0035423)
Supplement: Table S3 — Complete list of differentially expressed miRNAs. (PDF) [file pone.0035423.s004.pdf]

miRNA ID	Log Fold Change 402-91/ET vs 402-91	P.Value	adj.P.Val	Chr. localization
hsa-miR-10b	-2,6992146	2,18E-10	8,6E-09	2
hsa-miR-99b	-2,0506783	2,25E-09	0,000000071	19
hsa-miR-183	-1,9504999	0,000000139	0,00000219	7
hsa-let-7e	-1,6268365	1,07E-08	0,000000242	19
hsa-miR-125a-5p	-1,5703951	0,000000005	0,000000132	19
hsa-miR-96	-1,4845188	2,14E-11	1,13E-09	7
hsa-miR-192	-1,3879277	0,000000331	0,00000475	11
hsa-miR-324-5p	-1,38262	1,5E-14	2,34E-12	17
hsa-miR-126	-1,2172715	0,000000043	0,000000849	9
hsa-miR-542-3p	-1,1218224	0,0000542	0,000476	X
hsa-miR-22	-1,1035174	0,00077	0,00553	17
hsa-miR-210	-1,0736416	0,00626	0,0333	11
hsa-miR-31*	-0,9549717	0,00000985	0,0000973	9
hsa-miR-324-3p	-0,9420725	7,03E-08	0,00000123	17
hsa-miR-199b-5p	-0,9004626	0,000000938	0,0000124	9
hsa-miR-100	-0,7626396	0,0285	0,102	11
hsa-let-7c	-0,6976259	0,0000086	0,0000906	21
hsa-miR-10a	-0,5478597	0,0319	0,105	17
hsa-miR-24	-0,5285212	0,00000702	0,0000793	9,19
hsa-miR-196a	-0,5042506	0,000286	0,00226	17,12
hsa-miR-130a	-0,4375004	0,0189	0,0806	11
hsa-miR-425	-0,381082	0,0242	0,0934	3
hsa-miR-15a	-0,3402029	0,0275	0,101	13
hsa-miR-98	-0,314965	0,0103	0,0464	X
hsa-miR-146a	3,1725678	0,0000127	0,000118	5
hsa-miR-193a-3p	2,9453256	8,68E-12	6,85E-10	17
hsa-miR-663	1,4476179	0,000478	0,0036	20
hsa-miR-29b-1*	1,2944914	0,00000136	0,0000166	7
hsa-miR-7	1,2056001	0,000128	0,00107	9,15,19
hsa-miR-1915	1,0801968	0,0144	0,0631	10
hsa-miR-365	1,0710406	0,000815	0,0056	16,17
hsa-miR-320a	1,0589873	0,00842	0,0429	8
hsa-miR-29c	1,024654	0,021	0,0861	1
kshv-miR-K12-3	0,9701683	0,0212	0,0861	-
hsa-miR-21	0,9517495	0,000928	0,00611	17
hsa-miR-320b	0,9470102	0,00333	0,0211	1
hsa-miR-17*	0,7574456	0,0037	0,0225	13
hsa-miR-29b	0,7083394	0,0305	0,104	7
hsa-miR-19b	0,6436695	0,0101	0,0464	13,X
hsa-miR-19a	0,5917163	0,0056	0,0316	13
hsa-miR-20a	0,5484608	0,0254	0,0957	13
hsa-miR-92a	0,547663	0,0234	0,0924	13,X
hsa-miR-320d	0,5326467	0,00633	0,0333	13,X
hsa-miR-320c	0,5082135	0,00521	0,0305	18
hsa-miR-16-2*	0,4801475	0,031	0,104	3
hsa-miR-30d	0,4780745	0,00943	0,0457	8
hsa-miR-181b	0,3642324	0,00954	0,0457	1,9
