# Supplementary material for: A Systems Biology Approach to Characterize the Regulatory Networks Leading to Trabectedin Resistance in an In Vitro Model of Myxoid Liposarcoma
Source: PLoS One. 2012 Apr 16;7(4):e35423. doi: 10.1371/journal.pone.0035423 (PMC3327679; doi:10.1371/journal.pone.0035423)
Supplement: Table S5 — Complete list of predicted transcription factor binding sites enriched in the promoter region of up and downregulated gene in 402-91/ET cell lines. (PDF) [file pone.0035423.s006.pdf]

TFBS	Zscore	p-value	Expression 402-91/ET vs 402-91
REL	14,94	1,20E-09	up
SP1	12,92	1,20E-08	up
ELK1	12,82	3,80E-04	up
RELA	12,5	1,20E-05	up
MZF1_1-4	11,55	9,50E-05	up
ELF5	9,8	1,30E-03	up
TEAD1	9,5	8,80E-06	up
NF-kappaB	8,8	1,40E-05	up
Ar	8,4	3,30E-03	up
TLX1-NFIC	8,1	1,60E-03	up
MZF1_1-4	53,16	1,00E-02	down
SP1	48,39	7,50E-08	down
ZNF354C	36,35	9,80E-02	down
MZF1_5-13	35,11	2,60E-04	down
Arnt-Ahr	23,35	3,40E-04	down
ZEB1	22,01	1,10E-02	down
Roaz	21,77	9,90E-05	down
NFKB1	19,2	2,50E-05	down
Myf	19,04	4,50E-03	down
Pax4	17,49	8,60E-03	down
